# FPGA Modeling and Optimization of a SIMON Lightweight Block Cipher

**DOI:** 10.3390/s19040913

**Published:** 2019-02-21

**Authors:** Sa’ed Abed, Reem Jaffal, Bassam Jamil Mohd, Mohammad Alshayeji

**Affiliations:** 1 Department of Computer Engineering, Kuwait University, Safat 13060, Kuwait; reem.jaffal@ku.edu.kw (R.J.); m.alshayeji@ku.edu.kw (M.A.); 2 Department of Computer Engineering, Hashemite University, Zarqa 13115, Jordan; bassam@hu.edu.jo

**Keywords:** security, cipher, block cipher, encryption, lightweight block cipher, FPGA, power, energy, low-resource devices, SIMON

## Abstract

Security of sensitive data exchanged between devices is essential. Low-resource devices (LRDs), designed for constrained environments, are increasingly becoming ubiquitous. Lightweight block ciphers provide confidentiality for LRDs by balancing the required security with minimal resource overhead. SIMON is a lightweight block cipher targeted for hardware implementations. The objective of this research is to implement, optimize, and model SIMON cipher design for LRDs, with an emphasis on energy and power, which are critical metrics for LRDs. Various implementations use field-programmable gate array (FPGA) technology. Two types of design implementations are examined: scalar and pipelined. Results show that scalar implementations require 39% less resources and 45% less power consumption. The pipelined implementations demonstrate 12 times the throughput and consume 31% less energy. Moreover, the most energy-efficient and optimum design is a two-round pipelined implementation, which consumes 31% of the best scalar’s implementation energy. The scalar design that consumes the least energy is a four-round implementation. The scalar design that uses the least area and power is the one-round implementation. Balancing energy and area, the two-round pipelined implementation is optimal for a continuous stream of data. One-round and two-round scalar implementations are recommended for intermittent data applications.

## 1. Introduction

Recently, there was a rapid growth in applications based on low-resource devices (LRDs), which include radio-frequency identification (RFID), wireless sensor networks (WSNs), smart cards, wireless body area networks (WBANs), and the Internet of things (IoT) [1]. LRDs are designed for constrained environments where cost, power consumption, energy, and available resources are limited. As LRDs become ubiquitous in daily life, it is very important to protect the confidentiality of exchanged data. The challenge is to balance an adequate security level with the limited resources in LRDs. Special cipher implementation is required to optimize energy, power, and area while considering the constraints of the devices, such as ciphers or lightweight ciphers.

In general, ciphers in cryptography are algorithms responsible for encryption and decryption operations for an input message or plaintext by applying certain steps to generate the ciphertext. The algorithm of ciphers consists of three main sub-algorithms: encryption algorithm, decryption algorithm, and key-scheduling algorithm (also known as key-expansion) [2]. The key-scheduling or expansion algorithm is responsible for generating sub-keys used in various encryption/decryption steps. In most ciphers, key expansion is executed once for both decryption and encryption, while in others is executed separately for encryption and decryption such as the case in Advanced Encryption Standard (AES) and RIJNDAEL ciphers [1,2]. Based on the number of keys used on encryption/decryption algorithms, ciphers are categorized as asymmetric (public key) and symmetric (private key) ciphers [1].

Asymmetric ciphers are more expensive and complex in terms of computation [1,2]. The asymmetric algorithm uses two keys: public key and private key. The public key is public and can be accessible to everyone, while the private one is only known by one person and kept as a secret key. The public key and the private key are related to each other internally; however, they cannot be obtained from each other. Examples of popular asymmetric ciphers are RSA, DIFFIE-HELLMAN, ALGAMMAL, elliptic curve cryptography, and DSA [3].

Lightweight ciphers can be designed as symmetric ciphers [4], which use one shared private key employed in both encryption and decryption. Based on the number of processed bits and bytes, symmetric ciphers are categorized as block ciphers and stream ciphers. Block cipher is an encryption scheme for a fixed-size plaintext block [5]. Typically, blocks are 32 bits, 48 bits, 64 bits, or 128 bits. Block ciphers have three main parameters: key size, block size, and number of rounds [1]. Some researchers add the amount of logic in each round of the encryption algorithm as an additional parameter. Most of the commonly used symmetric ciphers are block ciphers, which include Data Encryption Standard (DES), 3DES, AES, BLOEFISH, and TWOFISH cipher [6]. On the other hand, stream ciphers perform encryption one bit or byte of plaintext input at a time to reduce design complexity [5]. Stream ciphers can be easily created by block ciphers. An example of the most popular stream cipher is RC4 [6]. Other examples include SALSA20, GRAIN, and TRIVIUM [7,8,9]. Based on the structure of the algorithms, block ciphers can be categorized into three groups: substitution permutation (SPN), Feistel stream, and Lai–Massey [10]. Compared with conventional blocks, lightweight block ciphers balance the following challenges: (1) minimal overhead (e.g., number of gates, memory footprint), because of the low-cost smart devices, (2) minimal power and energy consumption, and (3) sufficient security performance [10]. Cazorla et al. [11] stated that the lightweight cipher had smaller block sizes, such as 32 bits, 48 bits, or 64 bits. In addition, lightweight block ciphers simplified key scheduling, employing elementary operations per round, and increasing the number of rounds.

Cipher implementations targeted for low-resource applications are classified into software implementation and hardware implementation [1]. Hardware implementation is faster and lower in power and energy consumption, as compared with software implementation [4]. Hardware implementation is realized using application-specific integrated circuit (ASIC) technology or a field-programmable gate array (FPGA) platform [1]. Traditionally, an ASIC had better performance than an FPGA, but recent advances in FPGA technology reduced the speed gap [1,12]. FPGA provides more flexibility, re-configurability features, low-cost designs [13], and efficient resource utilization and hardware architecture [12]. Additionally, FPGA is well suited for implementing ciphers, as it offers many advantages, such as algorithm agility, upload, and modification in addition to the security features. Hardware implementation optimizes metrics, including throughput, area, power, and energy performance. For LRDs, power and energy are the most important metrics.

SIMON and SPECK, published by the National Security Agency (NSA), are highly optimized lightweight ciphers, which provide security and flexibility over a variety of configurations (different block and key sizes). Each of these ciphers demonstrates excellent performance in both hardware (in terms of area) and software (in terms of code size and memory usage), because of the flexible implementations [14]. A SIMON cipher is targeted for hardware and a SPECK cipher is targeted for software implementation [15]. While there are numerous published articles to optimize SIMON implementation for area and throughput, little attention is paid to optimizing power/energy, considered a fundamental issue in LRDs.

The novelty of this work is exercising various design options on SIMON FPGA implementations and modeling the implementation performance metrics with emphasis on energy in order to achieve the optimum design implementation. Reasonably accurate performance models are derived to better implement current and future cipher designs. The following steps are considered to achieve the aforementioned goal:

- Implement a basic SIMON design (scalar/non-pipelined) with one and multiple rounds. Scalar implementations are more appropriate for intermittent (non-continuous) data.
- Implement and examine pipelined design (pipelined) with one and multiple rounds. Pipelined designs are better suited to encrypt a continuous stream of data.
- Derive accurate performance models for throughput, area, power, and energy metrics based on the implementation results.
- Determine the best implementation for each performance metric with a particular area and energy (with slightly higher emphasis on energy), i.e., the most critical metrics in LRDs.

The rest of this paper is organized as follows: Section 2 reviews the related work in this area, including software and hardware implementation for lightweight ciphers. Section 3 discusses the proposed research methodology, the SIMON lightweight cipher algorithm, and the scalar and pipelined implementations. Section 4 summarizes the implementation results. Section 5 presents the discussions and guidelines. Finally, Section 6 details the conclusions and the recommendations for future work.

## 2. Related Work

Numerous studies examined SIMON and SPECK implementations on different software and hardware platforms, as compared with other block ciphers. This section presents an overview of these studies.

With regard to software implementation, Beaulieu et al. [16] implemented SIMON and SPECK ciphers on an 8-bit AVR microcontroller’s platform to achieve optimal performance. SIMON and SPECK were also compared with other block ciphers in the same AVR platform. SPECK demonstrated the best performance. Hosseinzadeh et al. [17] implemented lightweight block ciphers on an Atmega128 microprocessor, which were evaluated in terms of energy and memory performance. The block ciphers included KLEIN-80, TWINE-80, PICCOLO-80, SPECK64/96, and SIMON64/96. The results showed that SPECK64/96 was the best in terms of energy, followed by TWINE-80. In terms of memory consumption, the TWINE-80 block cipher was best and SPECK64/ 96 was ranked third. This study concluded that SPECK64/96 and TWINE-80 were the most suitable block ciphers for WSNs.

Several studies focused on hardware implementation using an ASIC flow. Beaulieu et al. [18] presented different ASIC implementations for SIMON and SPECK, including bit-serial, iterated, and partially and fully pipelined. The results concluded that SIMON had the highest efficiency when compared to other ciphers. Beaulieu et al. [14] discussed SIMON and SPECK implementations on ASIC hardware and 8-bit microcontroller software platforms. Comparisons with different lightweight block ciphers were provided, such as KATAN, KLEIN, MCRYPTON, PICCOLO, PRESENT, and AES. The ASIC results showed that PRESENT-80 achieved a throughput of 12.4 Kbps at 100 kHz within 1030 GE, while SIMON64/96 and SPECK 64/96 achieved higher throughput with less area (838 and 984 GE, respectively). Additionally, SIMON64/96 and SPECK64/96 provided 16 added bits of security. SIMON128/128 and SPECK128/128 required half of the AES area, which was better for hardware implementation. Beaulieu et al. [19] provided ASIC implementation results of different versions of SIMON and SPECK block ciphers, in terms of area and throughput. A comparison of different block ciphers (PRESENT, KATAN, KLEIN, PICCOLO, and AES) was also presented. The results showed that SPECK had a small ASIC implementation; however, SIMON had the smallest area of all investigated ciphers.

Other studies examined hardware implementations using an FPGA design flow. Beaulieu et al. [19] discussed FPGA performance comparisons of SIMON, SPECK, and PRESENT on low-cost Xilinx Spartan FPGAs. SIMON and SPECK demonstrated better area reduction in comparison to AES and PRESENT. Wetzels et al. [20] implemented several hardware architectural designs for a SIMON64/128 block cipher on a Xilinx Spartan-6 FPGA series platform. These designs included the round function, as well as iterative, loop unrolling, inner-round pipelining, outer-round pipelining, and mixed pipelining architectures. The issues and trade-offs between these designs were discussed. Mixed pipelining architecture had the greatest throughput, while the round function was optimal in terms of area. Performance results were demonstrated for throughput, area, and throughput-to-area. Feizi et al. [21] implemented a SIMON32/64 block cipher in an FPGA model, Virtex-5 XC5VFX200T, and presented the results. SIMON was found to be a very flexible algorithm, due to the range of block and key sizes it offers. It is suitable for RFID systems and WSNs. SIMON32/64 has a small block and key size, and, as a result, it is suitable for lightweight devices, where few resources are needed. The larger key and block size offer more levels of security. Aysu et al. [15] suggested that SIMON is a strong alternative to AES, because it provides an equivalent level of security with better area results. The smallest area was achieved for low-cost FPGA by SIMON, which required 36 slices on a Spartan-3 FPGA and 13 slices on a Spartan-6 FPGA. Gulcan et al. [22] proposed a flexible FPGA hardware architecture capable of using all SIMON configurations. The implementation results showed that the proposed architecture required 90 and 32 slices on Spartan-3 and Spartan-6 FPGAs, respectively. Wan et al. [23] proposed an ultra-low-power implementation of a SIMON cipher. A bit-serialized SIMON core with 32-bit plaintext and 64-bit key was implemented. The design was based on adiabatic circuits. The proposed architecture achieved a 27.5× higher energy efficiency (kilobit per second per Watt) at the expense of 18% less throughput, as compared to conventional implementations. Yang et al. proposed [24] the SIMECK cipher, which is similar to SIMON, and has the same rounds as SIMON. SIMECK has a slightly smaller area and power. However, SIMECK is more vulnerable to attacks, as several studies demonstrated including References [25,26].

In summary, SIMON exhibits superior hardware metrics, which makes it a good candidate for hardware implementation, especially on an FPGA platform, because of its advantages over an ASIC. While most of the implementations of SIMON targeted area optimization [15,19,22], it is clear that little research was invested in power and energy, which are significant to LRD design. The focus of the work in Reference [23] was circuit design, typically destined for custom design ICs or ASICs. Our work is based on exploring design options in FPGA implementations. Therefore, these are two different fields. Additionally, adiabatic circuits have major issues of strong dependency on parameter variations, voltage threshold, and logic family. To our knowledge, this is the first work we know of which presents analysis and modeling of SIMON metrics to achieve optimum design implementation, while focusing on energy and power performance metrics.

## 3. Methods

**Figure 1:**
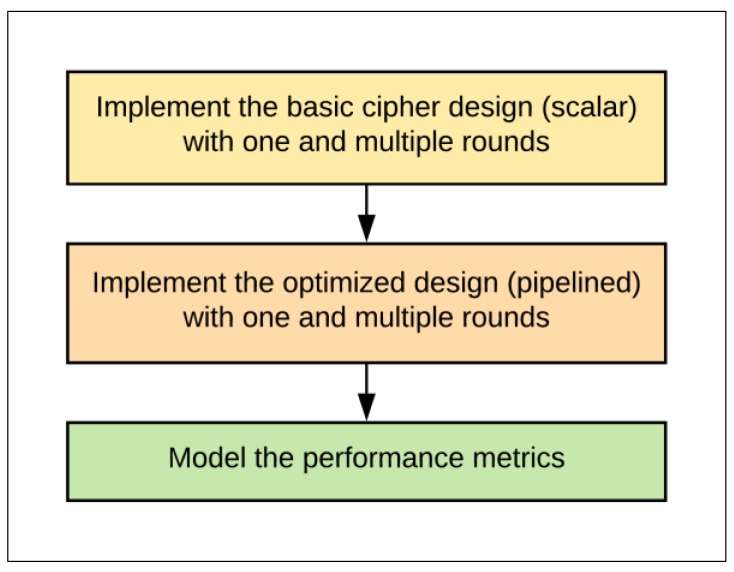
Research steps.

The research goal was to implement and optimize a SIMON block cipher in terms of throughput, area, power, and energy by considering different designs to determine the optimum implementation for a SIMON cipher. The optimum implementation balances area and power/energy consumption. The research steps to achieve this goal were as follows (see Figure 1):

- Implement the basic one-round SIMON scalar design to optimize the basic design.
- Implement scalar designs with multiple hardware rounds, e.g., 2, 4, 8, 16, or 32.
- Implement pipelined designs with one and multiple rounds.
- Measure and model the performance metrics (area, speed, power, and energy) for each step.
- Discuss recommendations for the optimum design.

**Figure 2:**
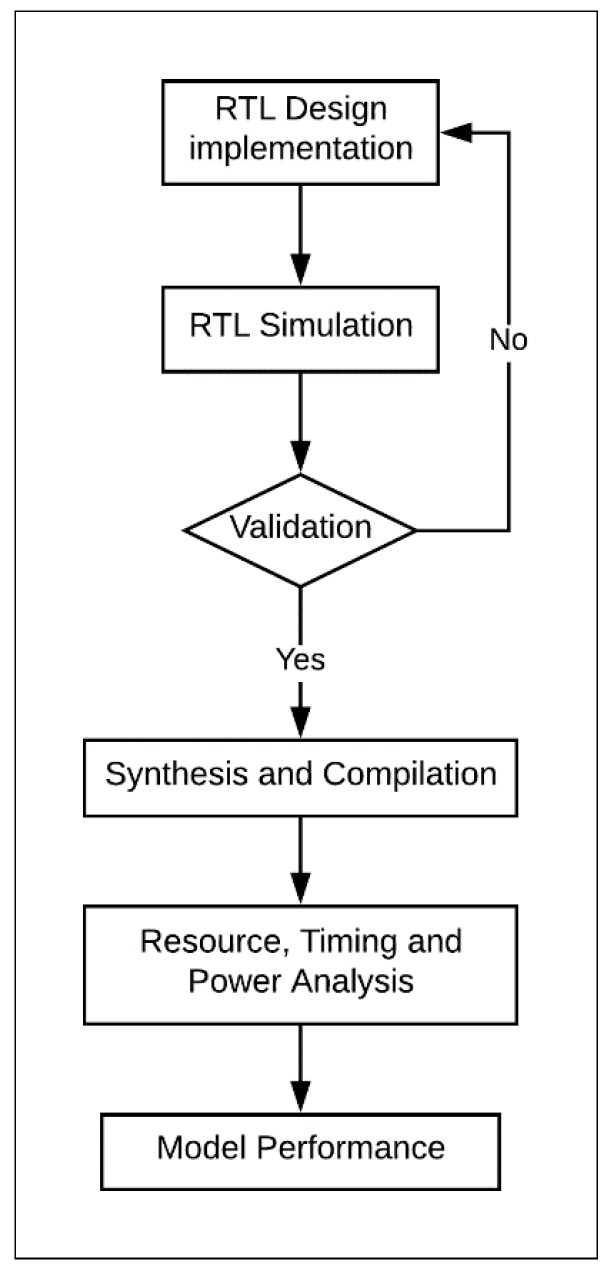
Design flow.

These steps can be achieved by executing the FPGA design flow shown in Figure 2. Similar FPGA design flows were used in other studies, including References [4,13,27,28,29,30,31]. The steps of the FPGA flow were as follows:

- The cipher was designed and implemented at the register transfer level (RTL) using Verilog^TM^, a hardware description language. The Verilog implementation was verified by dynamic simulations using ModelSim^TM^. ModelSim provides wave-files, which capture the node activity used to compute the power dissipation of the design.
- The design was synthesized and compiled using the Altera FPGA software package Quartus-II. The choice of FPGA family should not impact the results of the research. Mohd et al. examined several implementations of steganography algorithms in Altera and Xilinx FPGAs [32]. The study concluded that Altera and Xilinx provide similar trending results.
- For Quartus-II synthesis, timing constraints were used during the compiling process.
- The design underwent the following analyses using Quartus-II:
  - Timing analysis reported the maximum frequency of the design. The designs were compiled with clock constraints of 50 MHz.
  - Resource utilization analysis showed the number of logic elements (LEs) and the type (i.e., combinational, register logic, or both) used in the FPGA design [13]. LE is the smallest unit of logic in the Altera architecture; it is compact and facilitates efficient logic utilization. Each LE includes a four-input look-up table (LUT) and a programmable register. The LUT is a function generator that can implement any function of four variables [33].
  - Power analysis computed the average power of the design. The computed power is the dynamic core power that consists of combinational, register, and clock. The power required by node activities was extracted from the value change dump (VCD) files generated by ModelSim simulations. This approach to computing power was used in other works, such as References [4,13].
- Performance models for area, power, and energy were derived based on implementation results.

### 3.1. SIMON Algorithm

SIMON is one of the recently published lightweight block ciphers from the National Security Agency (NSA). The structure of the SIMON cipher is based on a Feistel network, which is an iterated cipher with an internal round function [34]. SIMON offers 10 different configurations depending on block and key sizes, which can provide numerous levels of security [22]; see the list in Table 1.

The SIMON block cipher is represented as SIMON2*n*/*mn*, where 2*n* is the block size, and *n* is the number of bits that define the word size, which could be 16, 24, 32, 48, or 64 bits. The key size is identified by multiplying the number of words in a key, indicated by the parameter *m*, by the word size, *n*, resulting in an *mn*-bit key length. For example, SIMON32/64 refers to 32-bit plaintext blocks, a 64-bit key size, and a 16-bit word.

SIMON32/64 was chosen as the cipher configuration in this study, because it supports block and key sizes appropriate for LRDs. Table 2 lists the SIMON notations used in this section. The parameters for SIMON implementation were as follows:

- Block size (2*n*): 32;
- Key size (*mn*): 64;
- Word size (*n*): 16;
- Key words (*m*): 4;
- Constant sequence (*z_j_*): *z*_0_;
- Cipher rounds (*T*): 32.

#### 3.1.1. Round Function

Encryption and decryption operations in SIMON2*n* apply the following on *n*-bit words [14]:

- Bitwise XOR, ⊕;
- Bitwise AND, &;
- Left circular shift, S^j^, by *j* bits.

**Figure 3:**
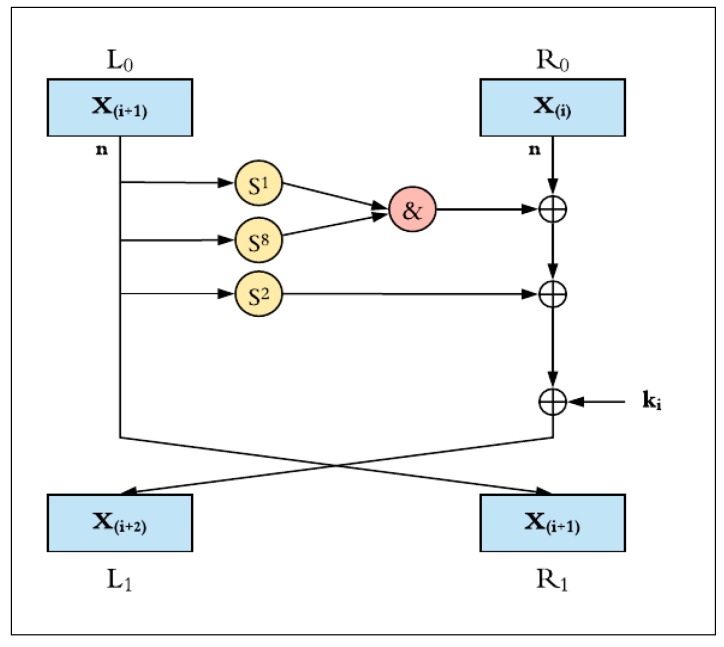
SIMON round function.

Figure 3 shows the round function of the SIMON cipher, where the input plaintext block (2*n*) is split into two equal words (each one is *n*-bit). In each round function, three circular shifts to the left (shift left one, shift left eight, and shift left two) and bitwise AND logic operations are performed on the left half block. The result is XOR-ed with the right half block and the round key. At the end of each round, the left half value is transferred to the right block and the generated value is written back to the left block. This round process is continuously repeated depending on the number of rounds within the implemented configuration. The SIMON round encryption function, *F*, is represented in Equation (1).

*F*(*x*,*y*,*k*) = (*y* ⊕ ((*S*^1^*x* & *S*^8^*x*) ⊕ *S*^2^*x*) ⊕ *k*), (1)

where *k* is the round key, *x* is the leftmost word of the cipher block, and *y* is the rightmost word.

#### 3.1.2. Key Schedule

The key schedule process applies the following basic operations on *m* words from the master key with *n*-bits for each:

- Bitwise XOR, ⊕;
- Right circular shift, S^−j^, by *j* bits.

The SIMON key schedule function takes the master key and generates a sequence of *T* key words (*k*_0_, *k*_1_, *k*_2_, …, *k*_*T*−1_), where *T* represents the number of rounds. There are three different versions of the key schedule function, depending on the block size and master key size, which can include two, three, or four words (i.e., *m* = 2, 3, or 4). The key schedule function performs two circular shift operations to the right (shift right one, and shift right three). The result is XORed with a fixed constant, *c*, and a constant sequence, *z_j_*. There are five sequences for the constant *z_j_*, which are version-dependent (i.e., *z*_0_, *z*_1_, *z*_2_, *z*_3_ and *z*_4_), as shown in Table 1.

**Figure 4:**
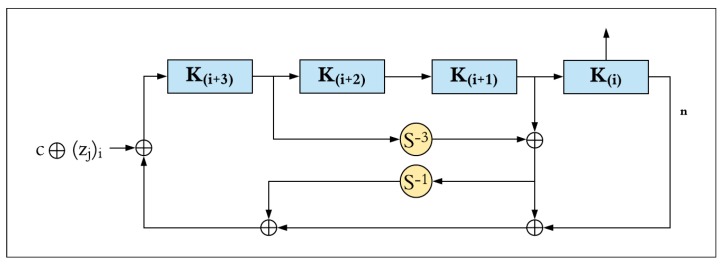
SIMON four-word key schedule.

Figure 4 illustrates the key schedule function of SIMON for four master key words (i.e., *m* = 4) of *n*-bits. These four sub-keys are generated on the first iteration of the key schedule function and are used as the first four round keys. A new round key is generated in each key schedule iteration. The block, *k_i_*, contains the round key required for the *i*-th round, where 0 ≤ *i* < (*T* − *m*) for the key schedule function. The most significant word *k*_*i*+3_ is circular shifted right by three (i.e., *S*^−3^), and then XORed with the word *k*_*i* + 1_. The result is circular shifted right by one (i.e., *S*^−1^). Finally, the result is XORed with the least significant word (*k_i_*) and the round constant (*c* ⊕ *z^i^*_j_). The value of *c* is equal to (2*n* − 1) ⊕ 3, which is a string of (*n* − 2) ones and two zeroes on the least significant two bits (i.e., *c* = 2*n* − 4 = 0xff … fc). The constant sequence, *z^i^*_j,_ is computed as the *i*-th bit of the *z_j_* sequence (from the most significant to least significant), where *i* is computed by (*i* − *m*) mod 62 for *m* ≤ *i* ≤ *T* − 1 and *j* is associated with each configuration, as shown in Table 1.

For SIMON32/64 with four key words (*m* = 4), the *c* constant, and the round constant sequence, *z_j_*, round keys are generated by Equation (2).

*k*_*i*+*m*_ = *c* ⊕ (*z_j_*)*_i_* ⊕ *k_i_* ⊕ (*I* ⊕ *S*^−1^) (*S*^−3^*k*_*i*+3_ ⊕ *k*_*i*+1_), (2)

where 0 ≤ *i* ≤ *T* − *m*, *T* = 32, *m* = 4, *c* = 2^16^ − 4, and *z*_0_ = 01100111000011010100100010111110110011100001101010010001011111 (represented in little-endian).

### 3.2. Scalar Design

This section details the scalar design implementation of the SIMON cipher. Firstly, it discusses the FPGA implementation of a basic scalar SIMON algorithm: the one-round implementation (iterative). Secondly, multiple hardware rounds are instantiated in the implementation to optimize area, power, and energy. A list of notations used in this section and the following sections is shown in Table 3.

#### 3.2.1. Basic Scalar Design for One Round (Iterative)

In the basic scalar design, one hardware round of the encryption unit is implemented as combinational logic connected to a single register and supplied with the proper round key. In the first clock cycle, the plaintext block is loaded into the register to perform the first cipher round. The result is then fed back to the circuit through the register. This process is repeated for *T* clock cycles, where *T* is the number of cipher rounds, as stated in Table 2. The ciphertext block is stored in the register. This design has two main features as follows:

1. Only one block cipher is encrypted at a time.
2. The number of clock cycles required to encrypt a single block cipher is equal to the number of cipher rounds (i.e., *T*) plus the number of cycles to load plaintext and output ciphertext (i.e., ***C_idle_***).

**Figure 5:**
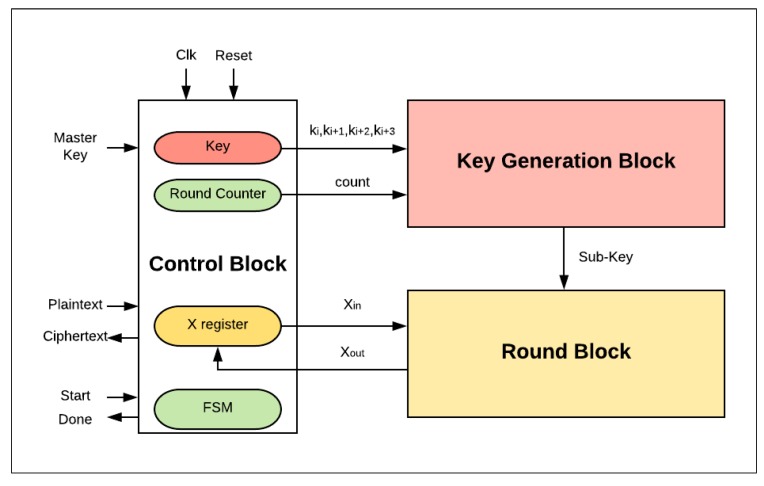
Basic SIMON field-programmable gate array (FPGA) design.

In the proposed design, the basic FPGA scalar design implementation of SIMON32/64 is described in Figure 5. Three main blocks are considered: the control logic, the round logic, and the key generation block.

- The control logic block is responsible for managing the external and internal activities of the system. It controls three main registers: key, round counter, and X register. Additionally, it organizes the sequence order of these activities’ functionalities through a finite-state machine (FSM). The encryption process begins with the assertion of a start signal. The plaintext is then loaded into the X register and the round counter is initialized to zero. The value of Key (master key) is also stored in specific sub-key registers in order to perform the key generation process. In the following cycles, the control block assigns sub-key and round counter values to the key generation block and Xin to the round logic block. Once the counter reaches its maximum value (the number of corresponding rounds has finished), the done signal is asserted by the control block to state that the encryption process is complete.
- The key generation block generates the sub-key required for the current round.
- The round block performs one hardware round operation and updates the X register. In the last clock cycle, the ciphertext value is saved in the X register.

#### 3.2.2. Scalar Design with Multiple Rounds (Loop Unrolling)

In the scalar design with multiple rounds, combinational logic is used to implement multiple hardware rounds instead of one round, as in the basic design. The loop of the basic design is unrolled to implement *r* rounds. If *r* is equal the number of rounds (i.e., *T*), a full loop unrolling design is the result. However, if *r* is less than the maximum number of rounds, partial loop unrolling is the result. The key schedules function is unrolled in the same manner. Hence, the number of iterations/rounds *R* required to encrypt one block of data decreases by factor of *r*. *R* is expressed by Equation (3).

*R* = *T*/r (3)

where *r* = 1, 2, 4, 8, 16, or 32.

The number of clock cycles required to encrypt a single block (*C_B_*) is obtained by Equation (4).

*C_B_* = *R* + *C_idle_*. (4)

**Figure 6:**
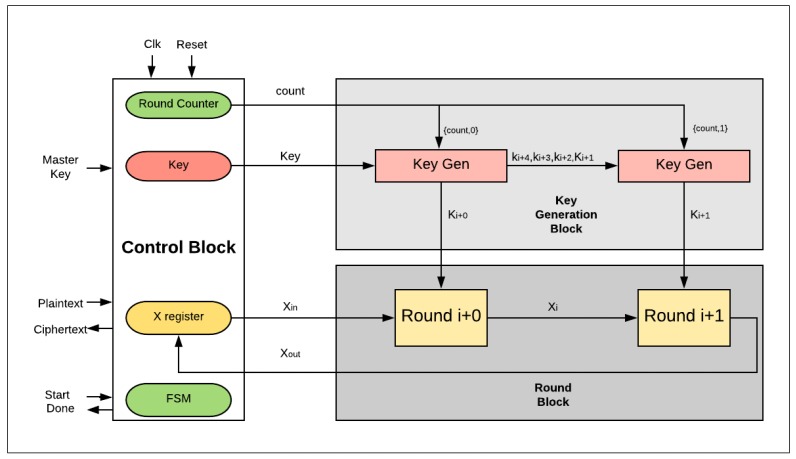
SIMON FPGA design with two rounds.

Figure 6 illustrates the design with two hardware rounds implemented into the SIMON cipher. The main differences between the two-round design and the basic (iterative) design are as follows:

- There are two hardware rounds in the two-round design, Round_i+0_ and Round_i+1_, which are executed simultaneously.
- There is a smaller counter in the two-round design: a 4-bit counter is required; in general, 2*^j^*-round design requires a (5-*j*)-bit counter.
- Dataflow for each round starts from the X-register to Round_i+0_, and then to Round_i+1_, returning to the X-register.
- Two sub-keys are generated each iteration instead of one sub-key, as in the basic design: sub-K_i+0_ and sub-K_i+1_ are required to feed Round_i+0_ and Round_i+1_.

### 3.3. Pipelined Design

**Figure 7:**
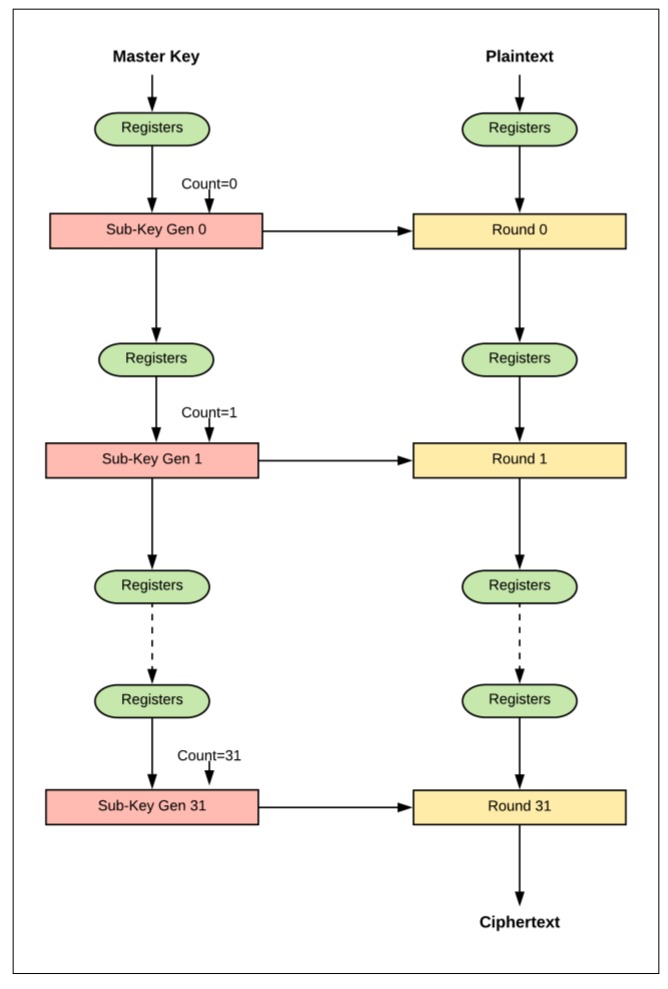
Implementation of one round per stage (pipelined design).

**Figure 8:**
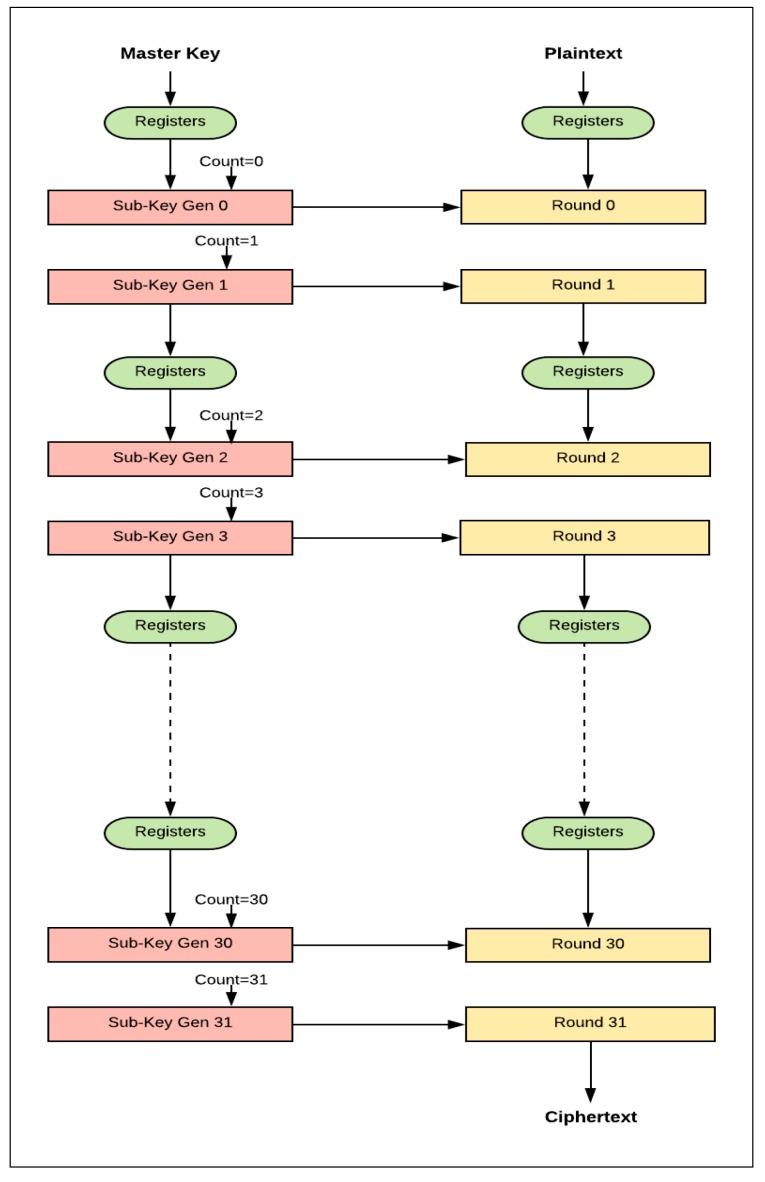
Implementation of two rounds per stage (pipelined design).

The 32-round (full loop unrolling) implementation of SIMON32/64 is extended to pipelined design by inserting registers between the unrolled rounds. The pipelined design, performing more than one task at a time, improves throughput. The pipeline design is better suited to process a continuous stream of data. The difference between the basic design (iterative) and the pipelined design is that new blocks of plaintext can be fed into the pipeline of each clock cycle, while, in the basic design, blocks are fed after (*T* + 2) clock cycles. *T* is the number of cipher rounds and two cycles are required to load in plaintext and output ciphertext. The pipelined design of SIMON32/64 encryption is shown in Figure 7. It consists of 32 stages with one round implemented in each stage, and registers are instantiated between stages. In each clock cycle, a new plaintext can be fed to the first stage. Key expansion functionality is implemented in the same pipelined method by instantiating registers between sub-key generation functions to feed the appropriate sub-key to the round function. Flavors of pipelined designs are implemented by varying the number of rounds (i.e., 1, 2, 4, 8, 16, and 32) in each pipelined stage. When the number of rounds per stage doubles, the number of stages is halved. Figure 8 shows the implementation of two rounds per stage.

## 4. Results

In this section, results for scalar and pipelined design implementations are presented and summarized. Implementation results include the following performance metrics: number of LEs, maximum frequency, and power and energy consumption.

Results are illustrated in tables and graphs. Tables include the exact values, while graphs only provide general trends of metrics. Metrics are normalized with respect to the one-round scalar design. The following notations are used to illustrate the results clearly:

- **S_r_** represents FPGA scalar implementation with *r* hardware rounds, where *r* = {1, 2, 4, 8, 16, 32}.
- **SP_r_** represents FPGA pipelined implementation with *r* hardware rounds per stage. The number of pipeline stages is equal to (32/*r*). As an example, SP_4_ has four implemented rounds per stage and has eight stages.

Table 4 lists the other notations used in this section.

### 4.1. Scalar Implementation Results

**Figure 9:**
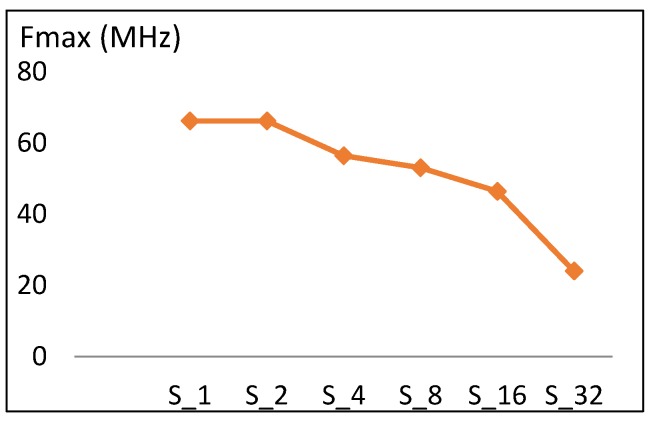
Frequency trend versus number of rounds for scalar implementations.

**Figure 10:**
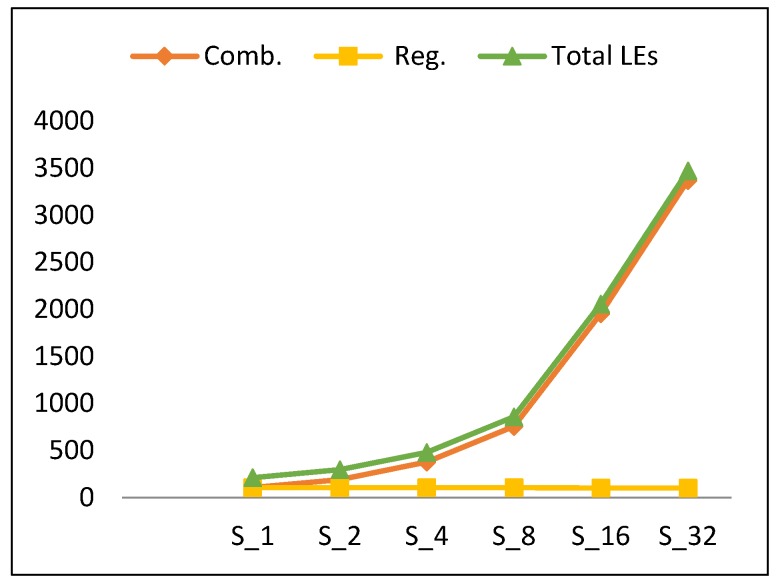
Resource utilization trend and its components versus number of rounds for scalar implementations.

**Figure 11:**
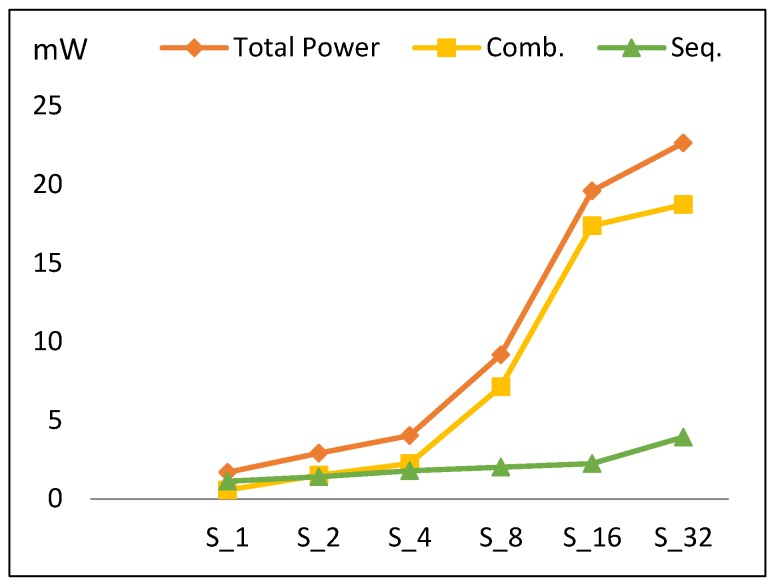
The power trend and its components for scalar implementations.

**Figure 12:**
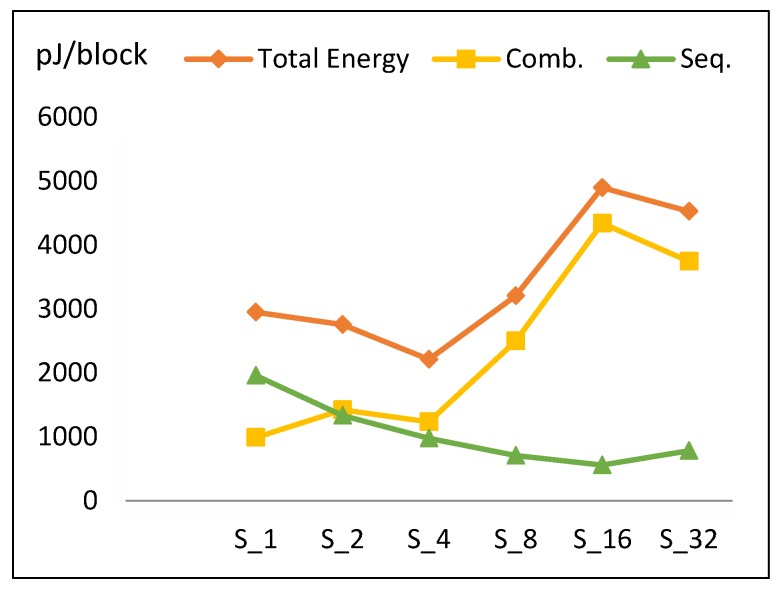
Energy trend and its components for scalar implementations.

This section presents the performance metric results for FPGA scalar design implementations illustrated in Section 3.2. Results are summarized in Table 5 and Figure 9, Figure 10, Figure 11 and Figure 12.

#### 4.1.1. Frequency

The maximum frequency versus number of rounds implemented for scalar designs is shown in Figure 9. Doubling the number of rounds leads to a decrease in the maximum frequency value. Frequency decreases by an average of 10 MHz, which is 16% of the S_1_ frequency. This reduction in frequency is due to the increase in the length of timing paths as more rounds are implemented in one cycle.

From the values in Table 5 and Figure 9, the frequency trend (*f*) is modeled using Equation (5), with an average model error of 10%.

*f* (S_2k_) = *f* (S_k_) − 0.16 *f* (S_1_), (5)

where *f* (S_1_) = 66 MHz, S_r_ = S_2k_, and k = [1, 2, 4, 8, 16].

#### 4.1.2. Resource utilization

Table 6 illustrates the resource utilization based on the type of LUTs, which can be 4-input functions, 3-input functions, 2-or-less input functions, and register only. Also, in this section, we present analysis of resources in terms of combinational LEs and register LEs. Register LEs include “register only” LEs and “combinational with a register” LEs. The latter analysis facilitates better understanding of power results.

Figure 10 shows the number of LEs versus the number of rounds implemented in the hardware for scalar designs. The number of LEs (which indicates the utilized resources) increases by an average of 78% when the number of rounds is doubled. To understand the source of this increase, the different types of LEs are identified, including combinational LEs and register LEs. Figure 10 also plots the LE components. Clearly, combinational LEs exhibit growth, while register LEs are constant, with respect to an increase in the number of implemented rounds. The growth component of LEs increases by 102% when the number of rounds is doubled. This is because the synthesis tool minimizes and shares the logic cones efficiently [13] and reuses the combinational logic. The number register LEs, however, is constant for each hardware implementation, except for the counter register, as it decreases by one bit when doubling the number of implemented rounds. Hence, the trend for the number of LEs (*LE*) is modeled using Equation (6) with an average model error of 9.4%.

*LE* (S_2k_) = *LE* (S_2k_ (Comb)) + *LE* (S_1_ (Register)) = 2.02 *LE* (S_k_ (Comb)) + *LE* (S_1_ (Register)), (6)

where *LE* (S_1_ (Comb)) = 105, *LE* (S_1_ (Register)) = 105, S_r_ = S_2k_, and k = [1, 2, 4, 8, 16].

#### 4.1.3. Power

Figure 11 describes the total power dissipation versus the number of implemented hardware rounds. Power increases by an average of 73% when the number of implemented rounds is doubled. Based on the type of circuit, there are two main components of power: sequential and combinational. Figure 11 also illustrates the contributions of combinational and sequential power. The following conclusions are drawn:

- Combinational power (which estimates the combinational logic power) increases by 105% when the number of rounds is doubled. The increase in the combinational power is due to an increase in implemented logic, as well as an increase in glitch power [35].
- Sequential power (which estimates the sequential logic, i.e., register, control block) increases by an average of 30%.
- The main reason for the total power increasing is the combinational power.
- The power trend shows a slight increase for S_32_ power when compared to the overall increasing average for other scalar designs. This is due to the reduction in combinational power growth at this point, as S_32_ is optimized by the synthesis tool to reduce wiring, thereby reducing routing power.

The power trend (*p*) is modeled according to Equation (7) with an average model error of 13.2%.

*P* (S_2k_) = *P* (S_2k_ (Comb)) + *P* (S_2k_ (Seq)) = 2.05 *P* (S_k_ (Comb)) + 1.30 *P* (S_k_ (Seq)), (7)

where *P* (S_1_ (Comb)) = 0.6 mW, *P* (S_1_ (Seq)) = 1.1 mW, S_r_ = S_2k_, and k = [1, 2, 4, 8, 16].

#### 4.1.4. Energy

“Energy per block” is computed by multiplying “average power” by the “time to process one block”, which is expressed in Equation (8).

*T_block_* = *C_B_* × *T_cycle_* = (*R* + *C_idle_*) × *T_cycle_* = (*T*/*r* + 2) × *T_cycle_*. (8)

The two cycles in Equation (8) are required to load in plaintext and output ciphertext (i.e., *C_idle_*).

Energy per block versus number of implemented hardware rounds is illustrated in Figure 12. Doubling the number of rounds decreases energy from S_1_ to S_4_. S_4_ has the least energy dissipation (optimum design). Energy increases from S_4_ until it reaches its maximum value at S_16_ and slightly decreases again at S_32_.

To understand the behavior of the energy curve, the following facts are required:

- Increasing the number of implemented rounds increases combinational power and (to a lesser extent) sequential power, as shown in Figure 11.
- Increasing the number of implemented rounds, *r*, decreases the time to process one block (*T_block_*).

The synthesis tool performs better routing optimization in some implementations, resulting in noticeably less routing power.

Figure 12 shows the contribution of energy components (combinational and sequential) to the total energy. Based on the aforementioned facts, the following is found:

- Combinational energy in general increases 42% when the number of implemented rounds doubles. Routing power in S_16_ is not optimized, as compared to S_32_ and S_8_.
- Sequential energy slightly decays as the number of implemented rounds is doubled.
- Since energy is estimated by multiplying power with time to encrypt the block, and power and time exhibit different behavior with respect to *r*, the energy trend has a V-like curve, as seen in Figure 12.
- The highest energy consumption value at S_16_ is due to the high combinational energy from the high routing power/energy with additional glitch power/energy [36]. The drop of energy at S_32_ is due to the drop in combinational energy, as the tools optimize better for larger logic.

The energy trend (*E*) is modeled using Equation (9), with an average model error of 11.7%.

*E* (S_2k_) = *E* (S_2k_ (Comb)) + *E* (S_2k_ (Seq)) = 1.42 *E* (S_k_ (Comb)) + 0.73 *E* (S_k_ (Seq)), (9)

where *E* (S_1_ (Comb)) = 850 pJ, *E* (S_1_ (Seq)) = 1990 pJ, S_r_ = S_2k_, and k = [1, 2, 4, 8, 16].

### 4.2. Pipelined Implementation Results

**Figure 13:**
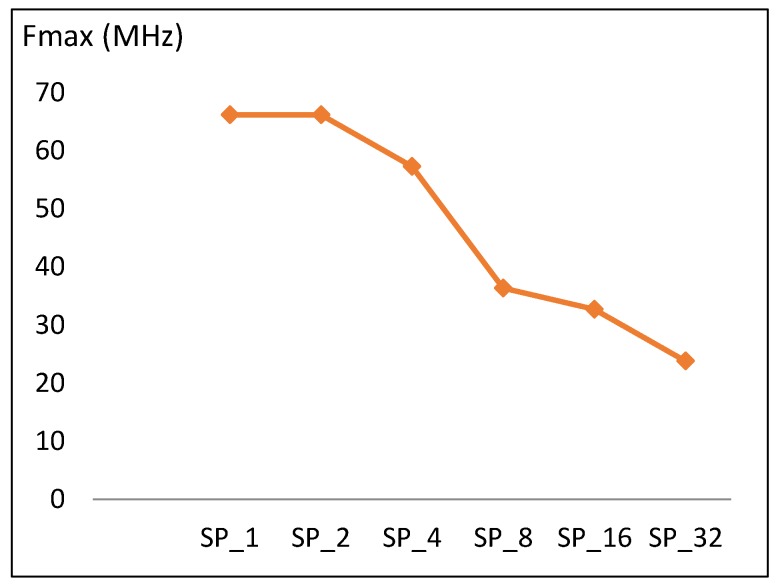
Frequency trend versus number of rounds for pipelined implementations.

**Figure 14:**
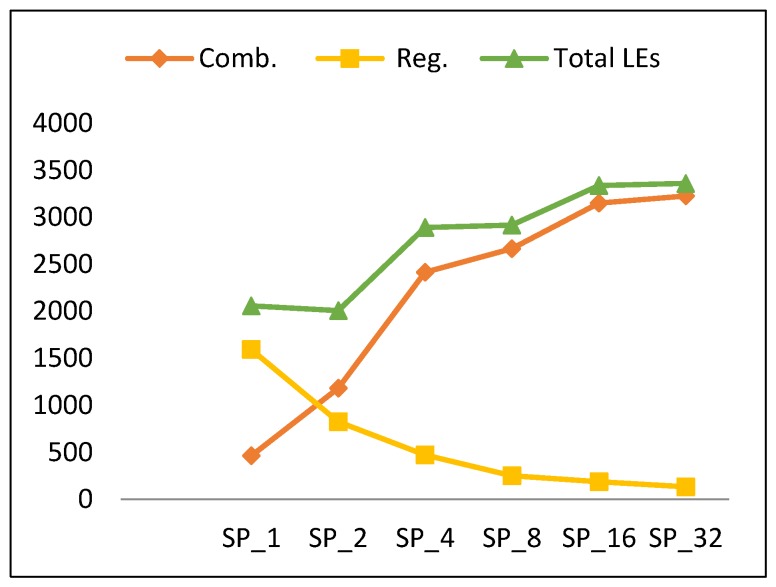
Resource utilization trend and its components versus number of rounds for pipelined implementations.

**Figure 15:**
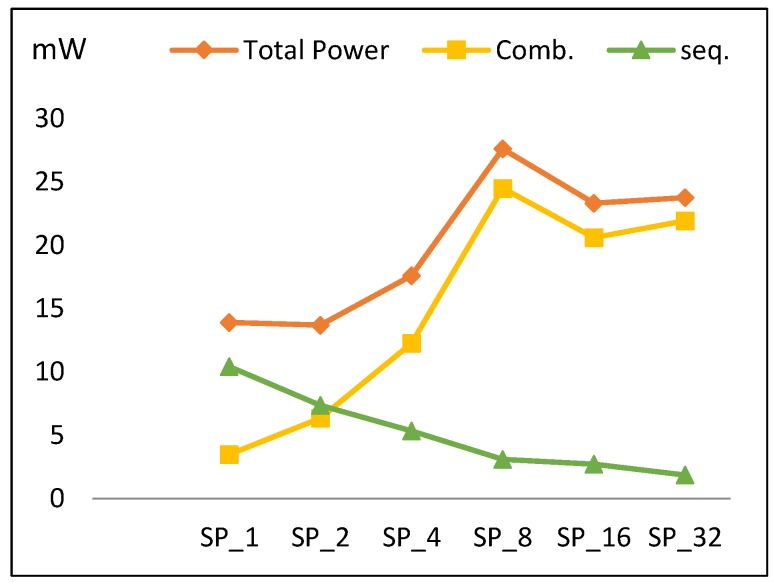
The power trend and its components for pipelined implementations.

**Figure 16:**
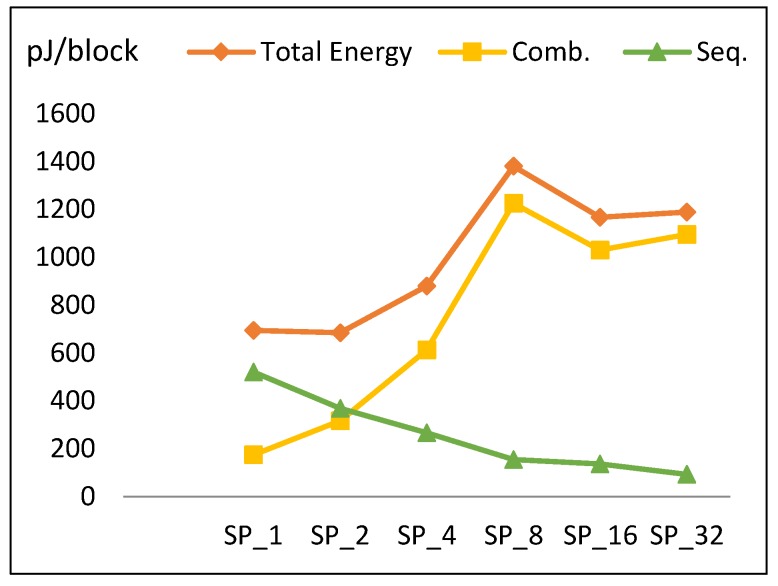
Energy trend and its components for pipelined implementations.

This section discusses the performance metric results for the FPGA pipelined design implementations discussed in Section 3.3. Results are summarized in Table 7 and Figure 13, Figure 14, Figure 15 and Figure 16.

#### 4.2.1. Frequency

Maximum frequency versus number of rounds implemented in a pipelined stage is shown in Figure 13. The trend is similar to the case of the scalar design, where doubling the number of rounds results in a drop in frequency of 10 MHz, which is equivalent to 16% of the SP_1_ frequency. Doubling the number of rounds implemented in one pipeline stage increases the critical timing paths and decreases frequency. The frequency trend (*f*) is modeled according to Equation (10) with an average model error of 9.8%.

*f* (SP_2k_) = *f* (SP_k_) − 0.16 *f* (SP_1_), (10)

where *f* (SP_1_) = 66 MHz, SP_r_ = SP_2k_, and k = [1, 2, 4, 8, 16].

#### 4.2.2. Resource Utilization

The resource utilization based on the type of LUTs is illustrated in Table 8. In this section, we present analysis of resources in terms of combinational LEs and register LEs. The latter analysis facilitates better understanding of power results as stated before.

Figure 14 shows the number of LEs versus the number of rounds per pipeline stage. There is a slight increase in LEs (an average of 15%) when doubling the number of rounds, except for SP_2_. To better understand this trend, Figure 14 also clarifies the source of the number of LEs as combinational-only LEs and register LEs. The number of combinational LEs increases when doubling the number of rounds per stage (similar to the scalar design), while the total number of register LEs decreases. This can be justified by the fact that total number of register LEs increases with the number of stages, as more registers are inserted between stages. SP_1_ has the largest number of registers, as it has the largest number of stages and pipeline partial results every round [13].

Generally, combinational LEs grow by an average of 40%. The total number of register LEs decays by an average of 42%. Clearly, from Table 7 and Figure 14, the number of LEs (*LE*) for SP_2k_ is modeled using Equation (11) with an average error of 15.4%.

*LE* (SP_2k_) = *LE* (SP_2k_ (Comb)) + *LE* (SP_2k_ (Register)) = 1.4 *LE* (SP_k_ (Comb)) + 0.58 *LE* (SP_k_ (Register)), (11)

where *LE* (SP_1_ (Comb)) = 800, *LE* (SP_1_ (Register)) = 1594, SP_r_ = SP_2k_, and k = [1, 2, 4, 8, 16].

#### 4.2.3. Power

The power trend across pipelined implementations is shown in Figure 15. Initially, power slightly decreases to its minimum value at SP_2_, and then increases until reaching its maximum value at SP_8_ before decreasing to SP_16_ and increasing to SP_32_. Overall, total power increases by an average of 14% when doubling number of implemented rounds. Figure 15 also plots power components (combinational and sequential power). The following is observed:

- As the number of rounds is doubled, combinational power grows by an average of 35%.
- As the number of rounds is doubled, sequential power decays by an average of 20% and the number of register LEs decreases.

The reason for the drop in the power at SP_16_ is due to combinational power, which is higher for SP_8_ because of the routing power. Combinational power reduction at SP_16_ relates to how the synthesis tool works in order to reduce connections and optimize the algorithm.

From Table 7 and Figure 15, the power trend (*P*) for SP_2k_ is modeled using Equation (12) with an average model error of 14%.

*P* (SP_2k_) = *P* (SP_2k_ (Comb)) + *P* (SP_2k_ (Seq)) = 1.35 *P* (SPk (Comb)) + 0.8 *P* (SPk (Seq)), (12)

where *P* (SP_1_ (Comb)) = 5 mW, *P* (SP_1_ (Seq)) = 10 mW, SP_r_ = SP_2k_, and k = [1, 2, 4, 8, 16].

#### 4.2.4. Energy

Energy to encrypt one block is computed by multiplying total power with the time required to encrypt the block. In the case of a full pipelined implementation, time to encrypt is one cycle, because every cycle of the pipeline completes one block. It is assumed that data are continuous (i.e., not intermittent). Energy per block versus number of rounds per pipeline stage is illustrated in Figure 16. The following is observed:

- The energy curve looks the same as the power curve with minimum energy at SP_2_. Energy increases gradually until reaching SP_8_, decays at SP_16_, and then increases to SP_32_.
- To better understand this trend, Figure 16 plots the energy components throughout pipelined implementations, which are combinational and sequential. Combinational energy increases by an average of 50%, while sequential decreases by an average of 15%. The growth in combinational energy is due to the glitch and interconnect power, while the decay in sequential energy is because of the decreasing number of flip-flops as the number of rounds per stage is doubled. The decay of combinational energy at SP_16_ is due to how the synthesis tool routes the connections and optimizes the design, as stated in Section 4.2.3.

From Figure 16, the energy trend (E) for SP_2k_ is modeled according to Equation (13) with an average model error of 14%.

*E* (SP_2k_) = *E* (SP_2k_ (Comb)) + *E* (SP_2k_ (Seq)) = 1.5 *E* (SP_k_ (Comb)) + 0.85 *E* (SP_k_ (Seq)), (13)

where *E* (SP_1_ (Comb)) = 170 pJ, *E* (SP_1_ (Seq)) = 520 pJ, SP_r_ = SP_2k_, and k = [1, 2, 4, 8, 16].

## 5. Discussion

In this section, the implementation results for scalar and pipelined designs presented in previous sections are analyzed and discussed in order to draw guidelines and conclusions. Moreover, the best design option or implementation for each performance metric is shown. Firstly, speed/timing and throughput metrics are discussed, followed by power and utilized resources (LEs) and the energy metric. Then, the optimum design is described. Finally, the implementations are discussed from a security perspective.

### 5.1. Speed and Throughput

**Figure 17:**
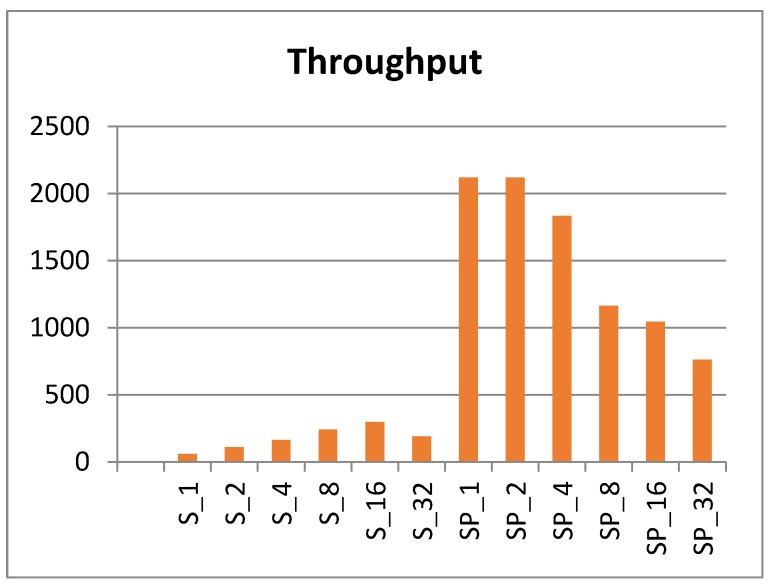
Throughput (encrypted-bits per second).

The fastest scalar and pipelined implementations are S_1_, S_2_, SP_1_, and SP_2_. These designs have the lowest logic in the clock cycle, the smallest period, and the fastest frequency. Throughput (encrypted-bits per second) is measured for each scalar and pipelined implementation, as shown in Figure 17.

Clearly, the pipelined implementations demonstrate better throughput (12 times higher than scalar). The best pipelined implementations are SP_1_ and SP_2_, while the best scalar implementations are S_16_ and S_8_. It is important to highlight that the pipelined design does not realize its full potential unless pipeline stages are full of encrypted blocks and, hence, are appropriate for applications with continuous streams of data blocks.

### 5.2. Power and LEs

**Figure 18:**
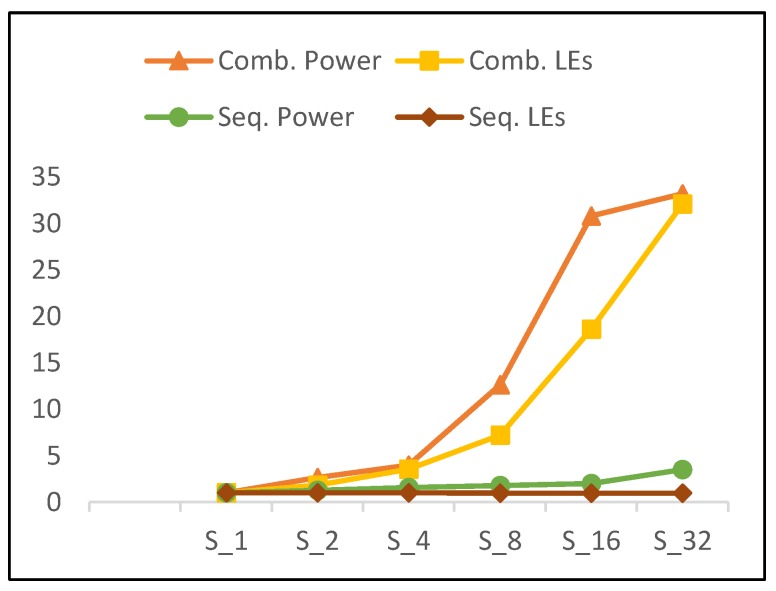
Combinational power vs. combinational logic elements (Les) and sequential power vs. sequential LEs for scalar implementations.

It is obvious that power and resource utilization (number of LEs) trends are related, as average growth with doubling hardware rounds is approximately the same. Figure 18 illustrates the trends for combinational power and combinational LEs of scalar implementations versus number of rounds. Figure 18 also illustrates the trend for sequential power and sequential LEs. Combinational power and number of combinational LEs exhibit the same increasing trend. Moreover, the growth in power (i.e., 2.05) is slightly higher than the growth of combinational logic (i.e., 2.02). Thus, power is not only affected by the number of LEs, but also by interconnects and glitch power [36].

As the number of rounds is doubled, sequential power increases, with no change in sequential logic. This is attributed to an increase in the activity of sequential circuits when more rounds are implemented in the hardware. The following results are observed for scalar implementations as the number of rounds is doubled:

- 102% growth in combinational LEs and 105% growth in combinational power.
- No change in sequential LEs and a 30% increase in sequential power.

**Figure 19:**
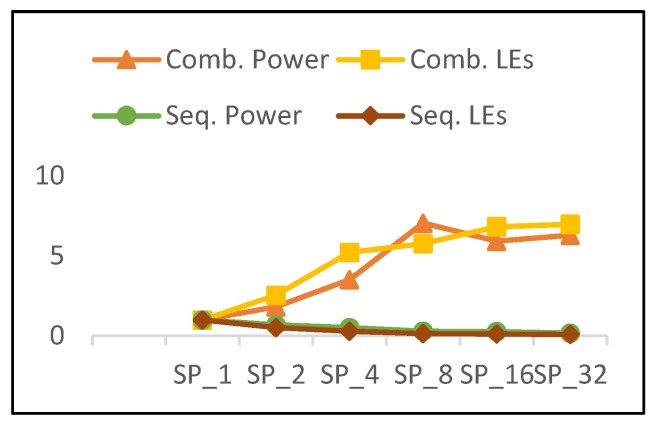
Combinational power vs. combinational LEs and sequential power vs. sequential LEs for pipelined implementations.

The combinational power and combinational LEs of pipelined implementations versus the number of rounds is shown in Figure 19, in addition to the sequential power and sequential LEs. The combinational power and combinational LEs trends increase in a similar manner; however, power growth is lower except for the case of SP_8_. Thus, power is not only affected by resource utilization, but also by interconnects and glitch power, as stated above. As the number of rounds is doubled, sequential power and sequential logic decrease, but the power decays less. The following results are observed for pipelined implementations as the number of rounds is doubled:

- 40% growth in combinational LEs and 35% growth in combinational power.
- 42% decay in sequential LEs and 20% decay in sequential power.

Clearly, there is a correlation between resources (represented by LEs) and power, since dynamic power is proportional to design area [13]. Although there is a relationship between power and the number of LEs, there are other factors contributing related to the following:

- Interconnect power and glitch power [36].
- The way the synthesis tool routes the connections and optimizes the designs.

**Figure 20:**
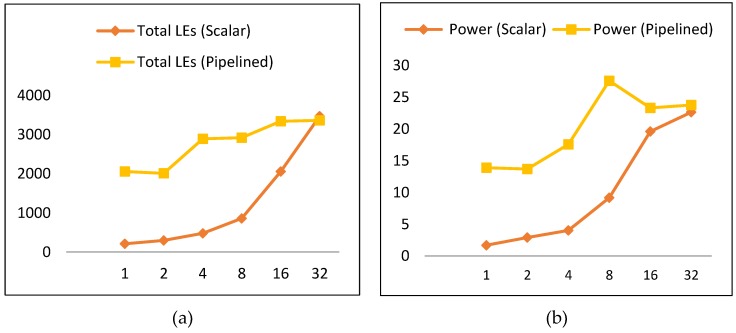
Power and LEs for scalar and pipelined implementations. (**a**) Total LEs for scalar and pipelined implementations; (**b**) total power for scalar and pipelined implementations.

When comparing scalar to pipelined implementations with respect to the number of LEs and power consumption, it is found that scalar designs are best in terms of power and resource utilization. Scalar implementations have lower LEs and power, as shown in Figure 20a,b, respectively. Scalar implementations consume, on average, 45% of the pipelined power and 39% of the pipelined LEs. Regarding scalar implementations, the best is S_1_ (the basic design), as it has the least power and the least number of LEs. Moreover, it consumes only 12% of the power of the best pipelined implementation (i.e., SP_2_), and the number of LEs is 10% that of SP_2_.

### 5.3. Energy

**Figure 21:**
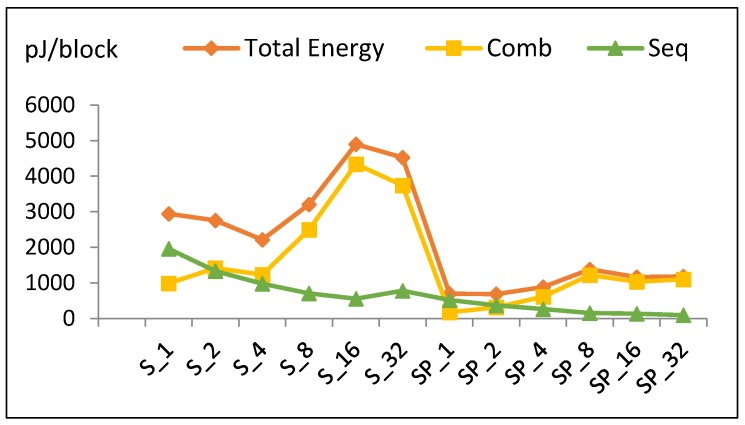
Energy per block for scalar and pipelined implementations.

Energy per block for all implementations (scalar and pipeline) is shown in Figure 21. This curve indicates the energy trend in order to easily identify the maximum and minimum energy implementations. The energy trend is different in scalar and pipelined designs. The combinational and sequential energy trends contribute to this total result.

**Figure 22:**
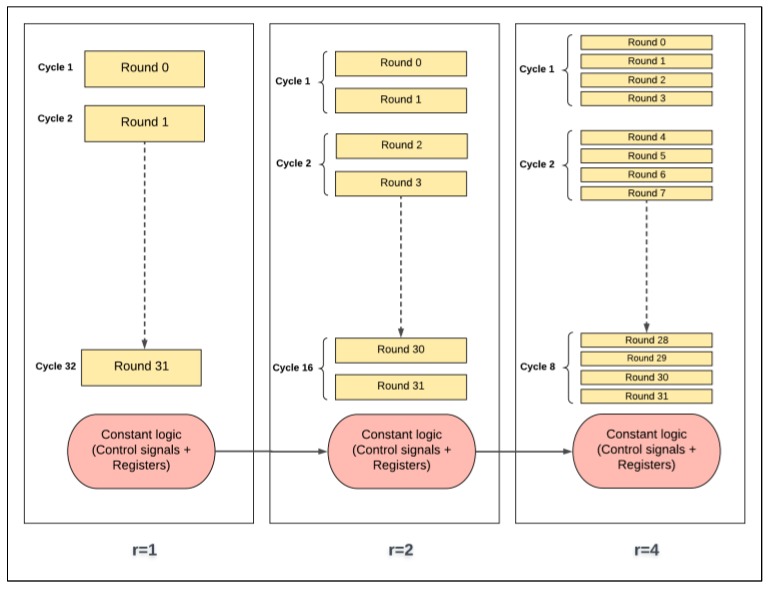
Control and round logic in scalar designs.

**Figure 23:**
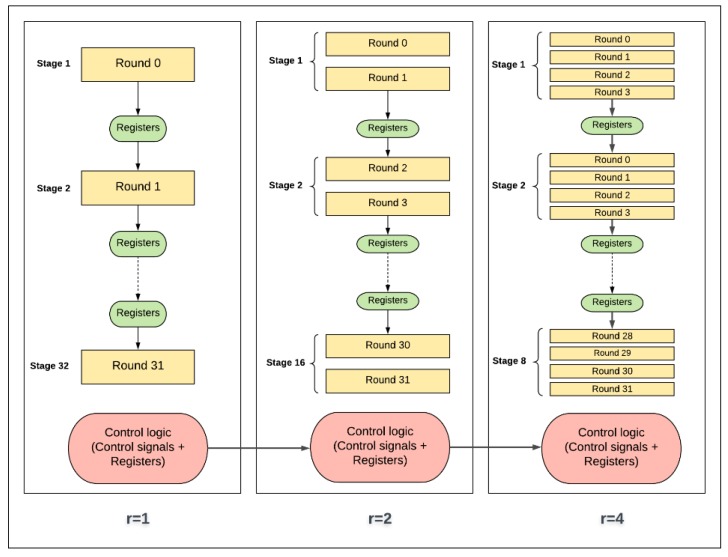
Control and round logic in pipelined designs.

The control and round logic concepts in scalar and pipelined implementations are presented as block diagrams in Figure 22 and Figure 23, respectively. It should be noted that control logic includes the control signals and registers that are not part of round and key scheduling logic.

In scalar one-round implementation, energy is computed by multiplying power by 32 cycles. In two-round implementation, energy is computed by multiplying power by 16 cycles (half the number of cycles of the previous implementation). Thus, instead of executing one round logic and one control logic (control signals and registers) per cycle as in the case of one-round implementation, two rounds of logic and one control logic are executed. As a result, doubling the number of rounds, *r*, decreases the number of cycles by a factor of two. This process results in the following:

- The control logic, which includes the control signals (e.g., clock, done, start signals, etc.) and registers (e.g., flip-flops, round counter), is executed 50% less as *r* is doubled. As the number of rounds is increased, the number of hardware iterations decreases. Hence, control logic energy decreases. Generally, clock and registers contribute less to energy with a higher number of rounds, which leads to energy savings. This is one source of the decreasing trend.
- Theoretically, the round logic should not be affected, because, as the number of rounds implemented in the hardware is doubled, cycles are halved. Yet, the following factors should also be taken into consideration when the number of hardware rounds, *r*, is doubled:
  - The synthesis tool can find more opportunity to optimize larger logic, as there is a better chance to reduce the logic. Thus, doubling the number of hardware rounds typically results in an area less than the summation of the two rounds. This is another source for the decreasing trend [37].
  - The logic becomes more complex with a larger number of rounds, due to many interconnections and levels. Thus, glitch and interconnect power and energy tend to increase [36].

The same control and round logic concepts are applied to pipelined implementation, as shown in Figure 23, with the addition of register logic. For one-round implementation, energy is computed by multiplying power with time to encrypt one block per stage. For example, consider the case of four-round implementation, where the cycle time increases as four rounds are processed in each stage. Hence, as the number of rounds, *r*, per stage is doubled, the clock period increases. This results in the following:

- The control logic is executed less as *r* is doubled. As the number of rounds per stage increases, the number of stages and the round counter decreases. Hence, control logic is simplified when doubling the number of rounds, and, as a result, power/energy decreases. This is one source of the decreasing trend in the pipelined implementation.
- The round logic is affected by two main factors as follows:
  - The synthesis tool tends to optimize larger logic better, as opportunities for sharing and minimizing logic cones increases. Therefore, as the number of rounds per stage is increased, power and energy decrease. This is another source for the decreasing trend.
  - Stage complexity increases as number of rounds per stage is doubled. As the level of complexity increases, interconnection, routing, and glitch power and energy increase. This is one source for the increase in the energy trend.
- The registers (i.e., flip flops) inserted between pipeline stages reduce by a factor of two when the number of rounds per stage is doubled, and the number of stages is halved. Thus, power and energy decrease. This is another source of the decreasing trend.

This analysis explains the mechanisms behind the energy trend. Energy also depends on the algorithm and the implementation. In general, pipelined designs dissipate lower energy compared to scalar designs. SP_2_ reports the lowest energy dissipation value closely followed by SP_1_ across all pipelined designs. For scalar designs, S_4_ has the lowest energy. Thus, the best implementation in terms of energy consumption is SP_2_, as it consumes 31% of the best scalar design (i.e., S_4_).

### 5.4. The Optimum Design

This section determines the best implementation of a lightweight cipher for LRDs. There are many performance metrics (i.e., throughput, power, area, and energy) to be considered, as discussed in the previous sections. In each performance metric, one implementation is optimum. Thus, the question is which metric is the most critical to consider. The answer will be the application to be used in lightweight ciphers. As stated before, lightweight block ciphers are suited for LRDs, as constrained environments supporting secure communication. With the continued minimization of transistor features and the need to increase battery lifetime, energy becomes the most critical issue for LRDs. Additionally, providing an adequate level of security without exceeding the resource limitation is critical. Therefore, energy and area are the most critical factors to be considered, with slightly higher emphasis on energy. The optimum metric is introduced in Equation (14), as in Reference [13], to compare different design implementations. This metric rewards the design implementations with minimum area and energy and emphasizes minimal energy.

Metric = *E*^µ^ × *LE*, (14)

where µ is the energy emphasis factor, and is evaluated using following values: 1.0, 1.2, 1.4, 1.6, 1.8, and 2.0.

**Figure 24:**
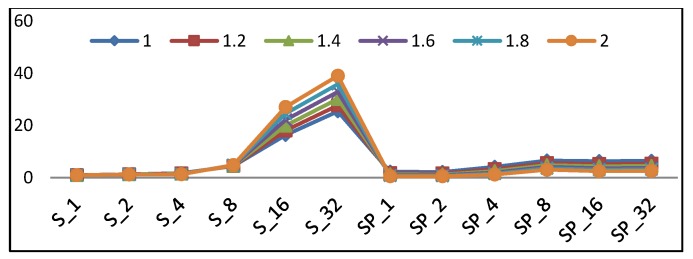
E^µ^ × LE, where µ is the energy emphasis factor.

Figure 24 shows the scalar and pipelined implementation trends using the metric in Equation (14) with different µ values. Changing the value of µ shows which design performs best at different energy levels. According to the curves in Figure 24, the following observations are drawn:

- With a higher emphasis on energy (µ > 1.5), the optimum implementation is SP_2_ followed by SP_1_.
- With a lower emphasis on energy (1 < µ < 1.5), the optimum implementation is S_1_ followed by S_2_.
- For scalar implementations, the optimum design is S_1_ followed by S_2_ and S_4_.
- In pipelined implementations, the optimum design is SP_2_ followed by SP_1_.

In general, balanced energy and area requirements lead to the following conclusions:

- Pipelined implementation performs better with a higher emphasis on energy and, as a result, is the best choice for low-resource/constrained devices.
- SP_2_ is best for the low-energy requirement, and S_1_ is the best for the low-resource requirement.
- The best implementations are SP_2_, SP_1,_ S_1_, S_2_, and S_4_.

The above conclusions are combined with the recommended usage for pipelined implementations started earlier (i.e., pipeline implementations are a better fit for applications with continuous blocks of data). Therefore, two-round pipelined implementation is optimum for applications with continuous streams of data, and one-round and two-round scalar implementations are recommended for intermittent data applications.

### 5.5. Implementations and Security

The presented implementations of the SIMON cipher do not alter the SIMON algorithm. The implementations exercise different design options to improve the performance metrics. Therefore, it is expected all implementations provide the same level of confidentiality security service. The only exception, however, is side-channel attack, which is implementation-dependent. Side-channel attack exploits the relationship between analog leakage (e.g., power) and the data manipulated by implemented design.

Bhasin et al. were successful in mounting side-channel attack and retrieval of the secret key in FPGA implementation of the SIMON cipher [38]. Bhasin et al. recommended loop unrolling to achieve higher side-channel attack resistance at minimal overhead [38]. For the presented pipelined implementations, all rounds are implemented in hardware, which provides optimum protection from side-channel attacks.

For scalar implementations, S_32_ unrolls all rounds and implements them in hardware, which is similar to pipelined implementations. S_1_ does not unroll rounds as it implements one round in hardware. Hence, S_1_ is most vulnerable to side-channel attacks amongst all implementations. Other scalar implementations unroll and implement a different number of rounds in hardware. Thus, when side-channel attack is a concern, the optimum scalar implementations might be slightly modified to be S_2_ and S_4._

## 6. Conclusions

This paper discussed the hardware implementations of SIMON lightweight cipher algorithm targeting secure communication in LRDs. Several scalar and pipelined FPGA implementations of the SIMON32/64 lightweight cipher were designed and examined with different numbers of hardware rounds per cycle.

Pipelined implementations performed better than scalar designs in terms of throughput performance by a factor of 12. The best pipelined implementations were SP_1_ and SP_2_, while S_16_ followed by S_8_ were the best scalar implementations. Additionally, the number of LEs and the measured power consumption trends were very similar. The best implementations in terms of LEs and power were scalar. Scalar implementations consumed, on average, 45% of the pipelined implementations power. Scalar LEs utilized 39% of pipelined LEs. As for scalar implementations, S_1_ was best; it consumed only 12% of the power of the best pipelined implementation (i.e., SP_2_) and the number of LEs for S_1_ was 10% that of SP_2_. In terms of energy dissipation, SP_2_ was best followed by SP_1_. Pipelined designs reported the lowest values for energy consumption compared to scalar designs. The best pipelined design (i.e., SP_2_) consumed only 31% of the best scalar design (i.e., S_4_).

Balancing energy and area, the optimum pipelined implementations were SP_2_ and SP_1_, while the best scalar implementations were S_1_, S_2_, and S_4_. The SP_2_ implementation is optimum for continuous streams of data, whereas S_1_ and S_2_ are recommended for intermittent data applications. When considering channel-side attack, S_1_ is not favorable.

This paper contributed to deriving accurate models for lightweight cipher performance metrics and providing general guidelines for future lightweight ciphers. This study also discussed opportunities to better implement future cipher designs with optimized energy and area, which is critical for LRDs targeting lightweight ciphers for the security purposes.

Future cipher designs should carefully examine small-round logic versus large-round logic. Small-round logic requires many rounds, *T*. Large-round logic implies few rounds. Historically, lightweight cipher designers choose small logic for better area and implement one round in hardware. However, this study showed that the one-round design was not optimal for energy. S_4_ and SP_2_ were best in terms of energy consumption. Our recommendation is to consider “fewer” larger rounds for future cipher design. This was also noted in Reference [37].

Future research could extend the work with different SIMON configurations and derive more general performance models for the SIMON cipher, depending on algorithm parameters (e.g., block size, key size, key words, and number of rounds). This would allow for the prediction of various performance metrics (including throughput, power, area, and energy) for each SIMON configuration. Extending the derived models to other platforms, such as ASIC technology, is also being considered.

## Figures and Tables

**Table 1:** The SIMON configurations and parameters.

Security Configuration	Block Size (2*n*)	Key Size (*mn*)	Word Size (*n*)	Key Words (*m*)	Constant Sequence (*z_j_*)	Rounds (*T*)
1	32	64	16	4	z0	32
2	48	72	24	3	z0	36
3	48	96	24	4	z1	36
4	64	96	32	3	z2	42
5	64	128	32	4	z3	44
6	96	96	48	2	z2	52
7	96	144	48	3	z3	54
8	128	128	64	2	z2	68
9	128	192	64	3	z3	69
10	128	256	64	4	z4	72

**Table 2:** SIMON cipher notations.

Notation	Description
⊕	Bitwise XOR
&	Bitwise AND
S^j^	Left circular shift, S^j^, by j bits
S^−j^	Right circular shift, S^−j^, by j bits
*n*	Word size
*2n*	Block size
*m*	Key words
*mn*	Key size
*i*	Round counter, where 0 ≤ *i* ≤ T − 1
*z_j_*	Constant sequence, where *j* = 0, 1, 2, 3, 4
*T*	Number of cipher rounds
F	Round function
*k*	Round key (sub-key)
*c*	Constant

**Table 3:** Equation notations.

Notation	Description
*r*	Number of rounds implemented in hardware
*R*	Number of iterations/rounds to encrypt one block
*T_cycle_*	Cycle time (i.e., clock period)
*T_block_*	Time to encrypt one block
*C_idle_*	Setup cycles to load input plaintext and output ciphertext
*C_B_*	Number of cycles to encrypt one block

**Table 4:** Results notations.

Notation	Description
*f*	Frequency of the design
*LE*	Resource utilization of the design
*P*	Power consumption of the design
*E*	Energy of the design

**Table 5:** Summary of the scalar implementation results. LE—logic element.

Scalar Implementation	Fmax (MHz)	LEs	Power (mW)	Energy (pJ/block)
S_1_	66. 2	210	1.68	2947
S_2_	66.19	294	2.9	2755
S_4_	56.42	479	4.02	2211
S_8_	53.11	858	9.16	3204.6
S_16_	46.41	2056	19.6	4900
S_32_	24.02	3469	22.64	4528

**Table 6:** Summary of the scalar implementation look-up table (LUT) results.

Scalar Implementation	LEs	LUT-4	LUT-3	LUT-2 or less	LUT RegOnly
S_1_	210	50	108	52	0
S_2_	294	121	103	68	2
S_4_	479	228	95	121	35
S_8_	858	554	191	98	15
S_16_	2056	1466	434	140	16
S_32_	3469	2410	718	324	17

**Table 7:** Summary of the pipelined implementation results.

Pipelined Implementation	Fmax (MHz)	LEs	Power (mW)	Energy (pJ/block)
SP_1_	66.2	2057	13.88	694.2
SP_2_	66.2	2008	13.69	684.4
SP_4_	57.32	2891	17.58	879
SP_8_	36.39	2918	27.60	1379.8
SP_16_	32.68	3338	23.32	1166
SP_32_	23.79	3361	23.76	1187.8

**Table 8:** Summary of the pipelined implementation LUT results.

Pipelined Implementation	LEs	LUT-4	LUT-3	LUT-2 or less	LUT RegOnly
SP_1_	2057	1400	83	477	97
SP_2_	2008	1379	263	349	17
SP_4_	2891	2001	461	347	82
SP_8_	2918	2031	562	277	48
SP_16_	3338	2264	766	282	26
SP_32_	3361	2422	591	325	23
